# Calmodulin binding proteins and neuroinflammation in multiple neurodegenerative diseases

**DOI:** 10.1186/s12868-022-00695-y

**Published:** 2022-03-04

**Authors:** Danton H. O’Day, Robert J. Huber

**Affiliations:** 1 Cell and Systems Biology, University of Toronto, Toronto, ON M5S 3G5 Canada; 2 Department of Biology, University of Toronto Mississauga, Mississauga, ON L5L 1C6 Canada; 3 Department of Biology, Trent University, 1600 West Bank Drive, Peterborough, ON K9L 0G2 Canada

**Keywords:** Neurodegeneration, Neuroinflammation, Calmodulin binding proteins, Calmodulin binding domains, Calcium, IQ motifs

## Abstract

Calcium dysregulation (“Calcium Hypothesis”) is an early and critical event in Alzheimer’s and other neurodegenerative diseases. Calcium binds to and regulates the small regulatory protein calmodulin that in turn binds to and regulates several hundred calmodulin binding proteins. Initial and continued research has shown that many calmodulin binding proteins mediate multiple events during the onset and progression of Alzheimer’s disease, thus establishing the “Calmodulin Hypothesis”. To gain insight into the general applicability of this hypothesis, the involvement of calmodulin in neuroinflammation in Alzheimer’s, amyotrophic lateral sclerosis, Huntington’s disease, Parkinson’s disease, frontotemporal dementia, and other dementias was explored. After a literature search for calmodulin binding, 11 different neuroinflammatory proteins (TREM2, CD33, PILRA, CR1, MS4A, CLU, ABCA7, EPHA1, ABCA1, CH3L1/YKL-40 and NLRP3) were scanned for calmodulin binding domains using the Calmodulin Target Database. This analysis revealed the presence of at least one binding domain within which visual scanning demonstrated the presence of valid binding motifs. Coupled with previous research that identified 13 other neuroinflammation linked proteins (BACE1, BIN1, CaMKII, PP2B, PMCA, NOS, NMDAR, AchR, Ado A2AR, Aβ, APOE, SNCA, TMEM175), this work shows that at least 24 critical proteins involved in neuroinflammation are putative or proven calmodulin binding proteins. Many of these proteins are linked to multiple neurodegenerative diseases indicating that calmodulin binding proteins lie at the heart of neuroinflammatory events associated with multiple neurodegenerative diseases. Since many calmodulin-based pharmaceuticals have been successfully used to treat Huntington’s and other neurodegenerative diseases, these findings argue for their immediate therapeutic implementation.

## Background

At least three common themes appear to underlie all well-studied neurodegenerative diseases: disruptive protein deposits, calcium dysregulation and neuroinflammation. The result is the dysfunction or loss of neurons in disease-specific regions of the brain. Increasing evidence argues that neuroinflammation is an early and critical event not only in Alzheimer’s disease (AD), where it has been extensively studied, but also in other neurodegenerative diseases including amyotrophic lateral sclerosis (ALS), frontotemporal dementia (FTD), Huntington’s disease (HD), Parkinson’s disease (PD), Lewy Body dementia (LBD), Batten disease (BD) and others [3, 6, 7, 46, 58, 60]. Neuroinflammation is a multistage process mediated mainly by microglia and astrocytes in the brain and influenced by external input (e.g., [20, 22, 23]. Chronic or unregulated neuroinflammation can lead to the uncontrolled release of pro-inflammatory factors that interfere with neuronal repair, cause synaptic impairment, mitochondrial dysfunction and disruption of the blood–brain barrier augmenting the neurodegenerative process [56].

Early and continued research first revealed the importance of dysregulated calcium levels in the events of AD leading to the Calcium Hypothesis [31, 32, 48]. The small calcium-binding protein calmodulin (CaM) is a primary effector of calcium function and works in turn by binding to and regulating CaM-binding proteins (CaMBPs) [53]. The Calcium Hypothesis was thus extended as the Calmodulin Hypothesis since CaM not only binds to and regulates CaMBPs critical to learning and memory but also proteins involved in the formation of amyloid plaques and tangles, hallmarks of AD [41]. Calcium dysregulation is also common to other neurodegenerative events [27]. CaM was subsequently shown to regulate many risk factor proteins, glutamate receptors (mGluR, NMDAR), ryanodine receptors, the adenosine A2A receptor, as well as other critical proteins linked to the onset and progression of AD and other neurodegenerative diseases [4, 9, 42–44]. The intimate role of CaMBPs in AD was recently reviewed reinforcing the role of CaM in binding to and regulating multiple key proteins adding further support for the Calmodulin Hypothesis of O’Day and Myre [41, 47]. However, one area that remains to be investigated is neuroinflammation.

**Fig. 1:**
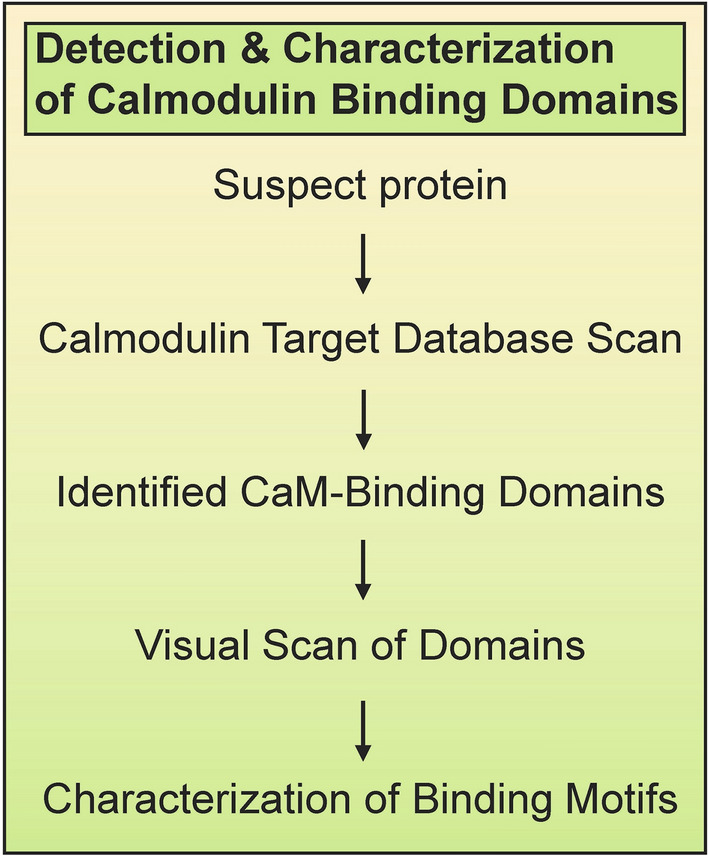
**Sequence of steps involved in evaluating calmodulin binding domains in suspect proteins**. Putative domains and binding motifs in suspect proteins were determined by performing a Calmodulin Target Database scan followed by a visual scan of the identified domains

**Fig. 2:**
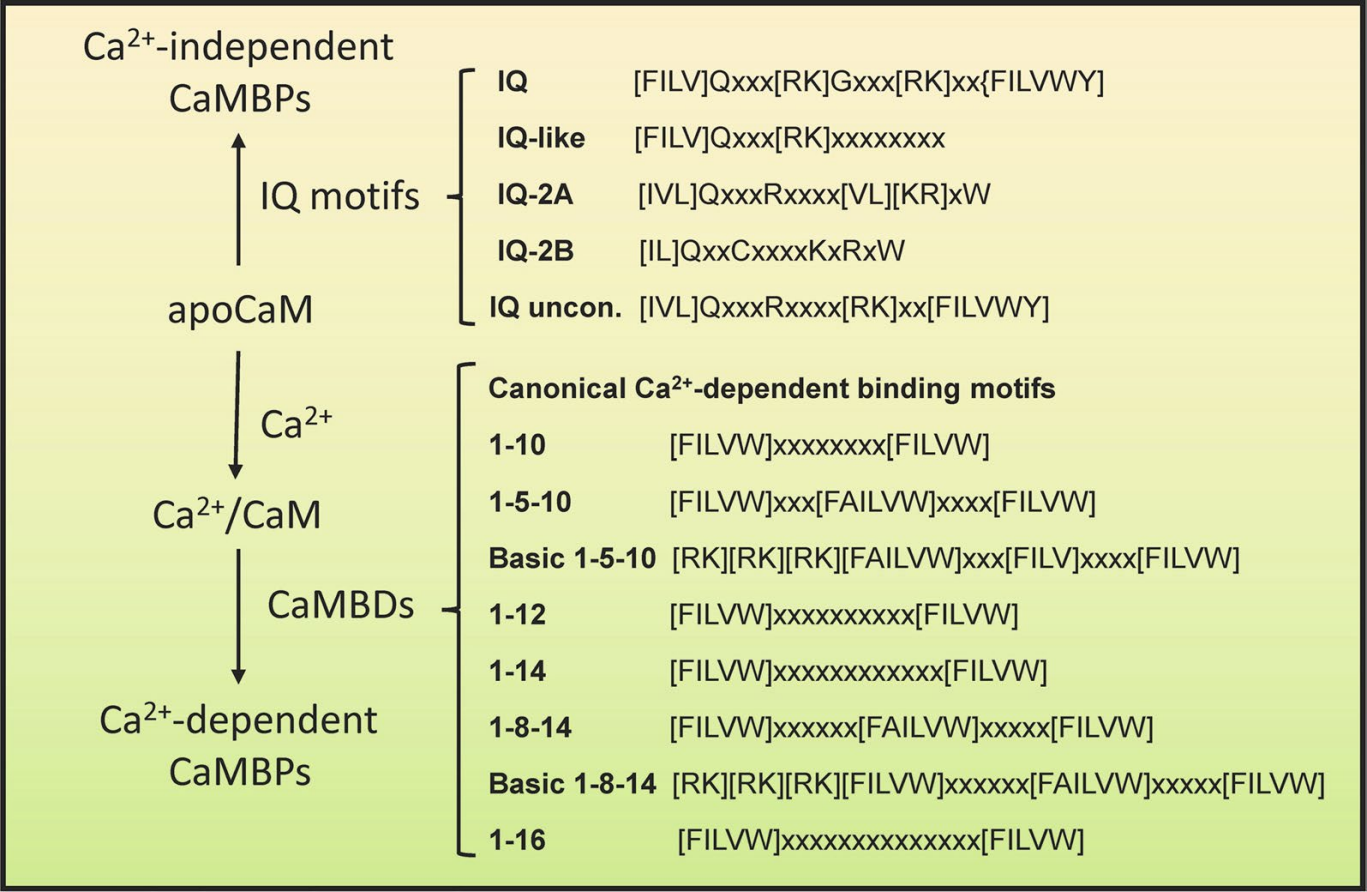
**Calcium-dependent and independent calmodulin binding motifs**. CaM, calmodulin; CaMBPs, CaM-binding proteins; CaMBDs, CaM-binding domains. Amino acids: A, Alanine; R, Arginine; Q, Glutamine; I, Isoleucine; L, Leucine; K, Lysine; F, Phenylalanine; W, Tryptophan; Y, Tyrosine; V, Valine; X, Any

Summarizing the work of others, Hampel et al. [22] noted that Genome Wide Association Studies (GWAS) identified multiple protein variants involved in AD neuroinflammation: triggering receptor expressed on myeloid cells 2 (TREM2), myeloid cell surface antigen CD33 (CD33), paired immunoglobin-like type 2 receptor alpha (PILRA), complement receptor type 1 (CR1), membrane-spanning 4-domains subfamily A (MS4A), clusterin (CLU), ATP-binding cassette subfamily A member 7 (ABCA7), and ephrin type-A receptor 1 (EPHA1). In addition, ATP-binding cassette subfamily A member 1 (ABCA1), chitinase-3-like protein I (CH3L1/YKL-40), and NACHT, LRR and PYD domains-containing protein 3 (NLRP3) are neuroinflammatory biomarkers linked to AD [1, 19, 50]. Several of these and other neuroinflammatory proteins have also been studied in LBD, HD, PD, and FTD [20, 37, 46, 55, 60, 64]. The critical function of ABCA7 in AD and FTD was recently reviewed [38]. Major risk factors for late-onset AD, CLU and CD33, are also involved in PD and multiple sclerosis (MS) [35, 54]. To gain insight into the potential role of CaM in neuroinflammation, we performed a literature search to determine if CaM-binding had been experimentally verified for any of these proteins and if any CaMBDs had been revealed. The remaining proteins, with sequences taken from the Uniprot database (www.uniprot.org), were subjected to a Calmodulin Target Database (http://calcium.uhnres.utoronto.ca/ctdb/no_flash.htm) search for the presence of putative CaMBDs [65] (Fig. 1). The Calmodulin Target Database uses Profile Hidden Markov Model algorithms and is recognized as the “dominant” method and “gold standard” for predicting CaM-binding, especially for proteins over 100 amino acids in length [39, 63]. Putative CaMBDs were then scanned visually to detect different binding motifs [41, 44, 51, 59] (Fig. 2). A priori, the greater the number of binding motifs argues the CaMBD has a higher chance of being a true binding domain. It should be noted that the Calmodulin Target Database primarily detects canonical, calcium-dependent CaMBDs leaving less common non-canonical and IQ motifs undetected. While non-canonical calcium-dependent CaMBDs can only be discovered through experimental means, calcium-independent IQ motifs can be detected through visual scanning of amino acid sequences thus setting the stage for their experimental validation.

## Main text

### Detection of putative calmodulin binding domains in proteins associated with neuroinflammation

**Fig. 3:**
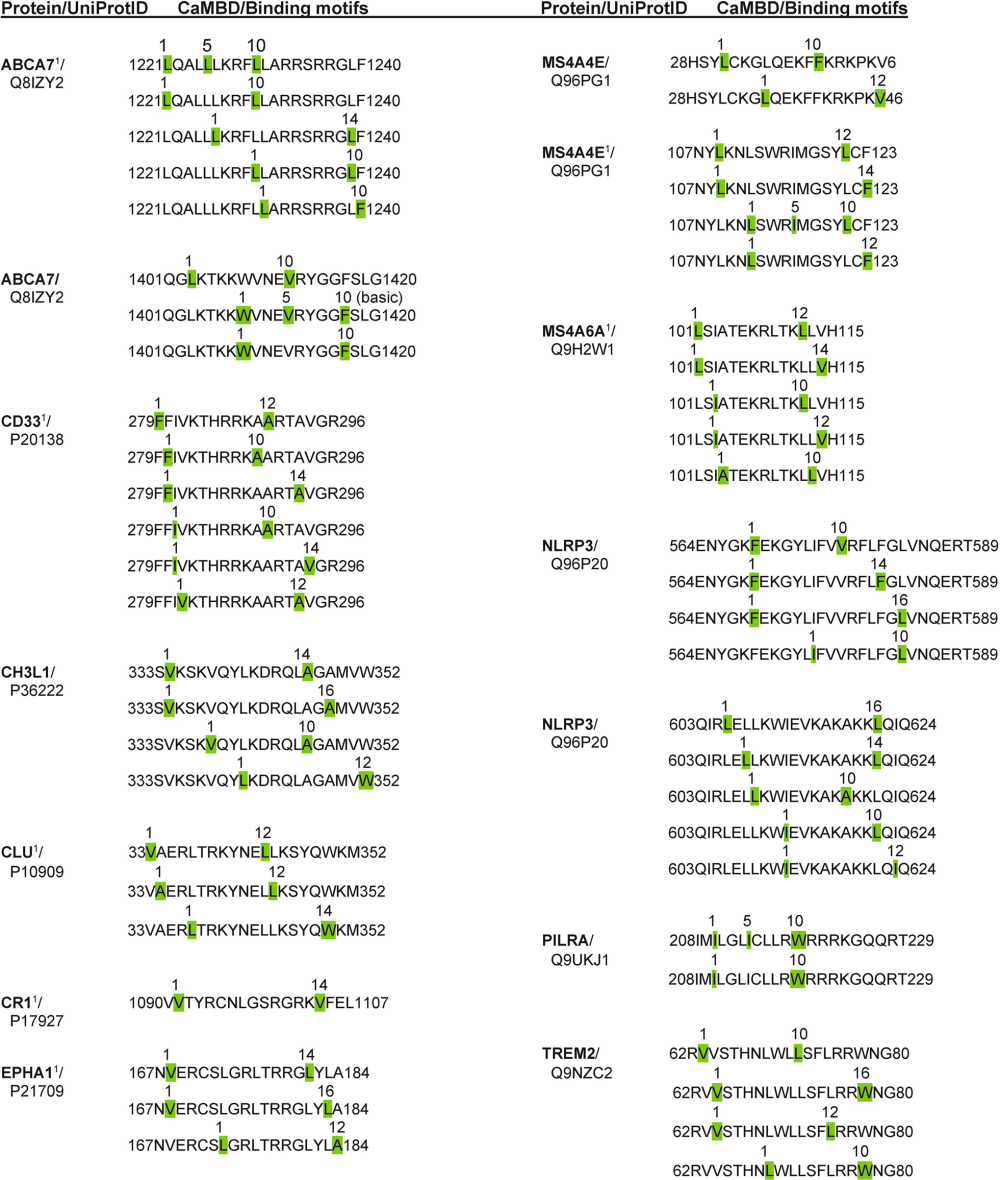
**Calcium-dependent calmodulin binding domains and motifs identified in human neuroinflammation proteins**. ABCA7, ATP-binding cassette transporter subfamily A member A7; CD33, Myeloid cell surface antigen CD33; CH3L1/YKL-40, Chitinase-3-like protein 1; CLU, Clusterin; CR1, Complement receptor type 1; EPHA1, Ephrin type-A receptor 1; M4A4E, Membrane-spanning 4-domains subfamily A member 4E; M4A6A, Membrane-spanning 4-domains subfamily A member 6A; NLRP3, NACHT, LRR and PYD domains-containing protein 3; PILRA, Paired immunoglobulin-like type 2 receptor alpha; TREM2, Triggering receptor expressed on myeloid cells 2. ^1^From O’Day, 2015; reanalyzed here. Green highlights: hydrophobic amino acids associated with binding motifs

Using the Calmodulin Target Database, calcium-dependent CaMBDs were identified in 11 proteins linked to neuroinflammation: ABCA7, CD33, CH3L I, CLU, CR1, EPHA1, MS4A4E, MS4A6A, NLRP3, PILRA, TREM2 (Fig. 3). Only one protein was found to contain calcium-independent IQ motifs (NLRP3). Details on the analyses are provided below.

#### Neuroinflammatory risk factor proteins

Our analysis revealed the presence of CaMBDs in multiple GWAS neuroinflammation risk factor proteins (Fig. 3). ABCA7 has a two CaMBDs. The previously detected CaMBD (1221LQALLLKRFLLARRSRRGLF1240) contains five calcium-binding motifs (three 1–10, one 1–5–10, one 1–14). The newly identified CaMBD (1401QGLKTKKWVNEVRYGGFSLG1420) [42] has two 1–10 and one 1–5–10 motif. ABCA1, a related ABC transporter involved in neuroinflammation, is a proven CaMBP with an experimentally verified 1–5–8–14 binding motif that protects the protein from degradation [26].

CLU and CD33 each possess a single CaMBD. The putative CaMBD in CLU (33VAERLTRKYNELLKSYQWKM352) contains three motifs (two 1–12, one 1–14) while CD33 contains six (two 1–10, two 1–12, two 1–14) (Fig. 3). CR1 has a CaMBD (1090VVTYRCNLGSRGRKVFEL1107) with a single 1–16 motif. The CaMBD of CR1 is almost identical to three other repeated 18-amino acid sequences within the protein at positions 207, 657 and 1560. While these other repeats are not recognized as binding domains by a scan via the Calmodulin Target Database, each of these sequences contains both 1–14 and 1–16 motifs found in the validated 1090-1107 CaMBD. EPHA1 has three motifs (1–12, 1–14, 1–16) within a single CaMBD (167NVERCSLGRLTRRGLYLA184). These results for CR1 and EPHA1 vary slightly from a previous study [42]. The risk factor MS4A proteins also have CaM-binding regions. The single CaMBD in MS4A6A (101LSIATEKRLTKLLVH115) has five different motifs (two 1–10, two 1–12, one 1–14), three more than previously detected [42]. Also, in contrast to previous results, a reanalysis of MS4A4E revealed two CaMBDs. The first (28HSYLCKGLQEKFFKRKPKV6) bears two motifs (1–10, 1–12) while the second (107NYLKNLSWRIMGSYLCF123) has four (two 1–12, one 1–14, one 1–5–10).

#### PILRA

PILRA contains one CaMBD (208IMILGLICLLRWRRRKGQQRT229) with two binding motifs (1–10, 1–5–10) (Fig. 3). While insights have been gained into receptor ligands for PILRA, nothing has been revealed about its downstream signaling events or the role of calcium and CaM in its function.

#### TREM2

The presence of a single CaMBD (62RVVSTHNLWLLSFLRRWNG80) within which multiple classical binding motifs are present (two 1–10, one 1–12, one 1–16) were found in human TREM2 (Fig. 3). This CaMBD falls outside of the AD TREM2 modifier mutation R62H (https://www.alzforum.org/mutations/trem2). Interestingly, T66M, a pathogenic mutation for an FTD-like syndrome, does fall within the domain but this amino acid change does not affect the CaMBD prediction [33]. On the other hand, this binding sequence is in the extracellular portion of the molecule and thus would be present in shed fragments. The role of extracellular CaM is detailed elsewhere but this may suggest that extracellular CaM should be considered in future studies on the role of this small calcium-binding protein in neurodegeneration [45].

#### NLRP3 inflammasomes

Two closely juxtaposed putative CaMBDs, CaMBD1 (564ENYGKFEKGYLIFVVRFLFGLVNQERT589) and CaMBD2 (603QIRLELLKWIEVKAKAKKLQIQ624) were revealed in human NLRP3 (Fig. 3). CaMBD1 contains four binding motifs (two 1–10, one 1–14, one 1–16) while CaMBD2 had 5 motifs (two 1–10, one 1–12, one 1–14, one 1–16). In addition, a single IQ-like motif (322WQKAERGDILLSSL335) was detected. Thus, it may be possible to use CaM antagonists to directly inhibit NLRP3 function to restore aspects of vascular dementia.

#### Chitinase 3-like I

While experimental studies remain to be carried out, a Calmodulin Target Database search and sequence analysis reveals that CH3L1 contains one potential 20-amino acid CaMBD (333SVKSKVQYLKDRQLAGAMVW352) with multiple motifs (one 1–10, one 1–12, one 1–14, one 1–16) (Fig. 3).

### The association of proven and putative calmodulin binding proteins with neuroinflammation

**Fig. 4:**
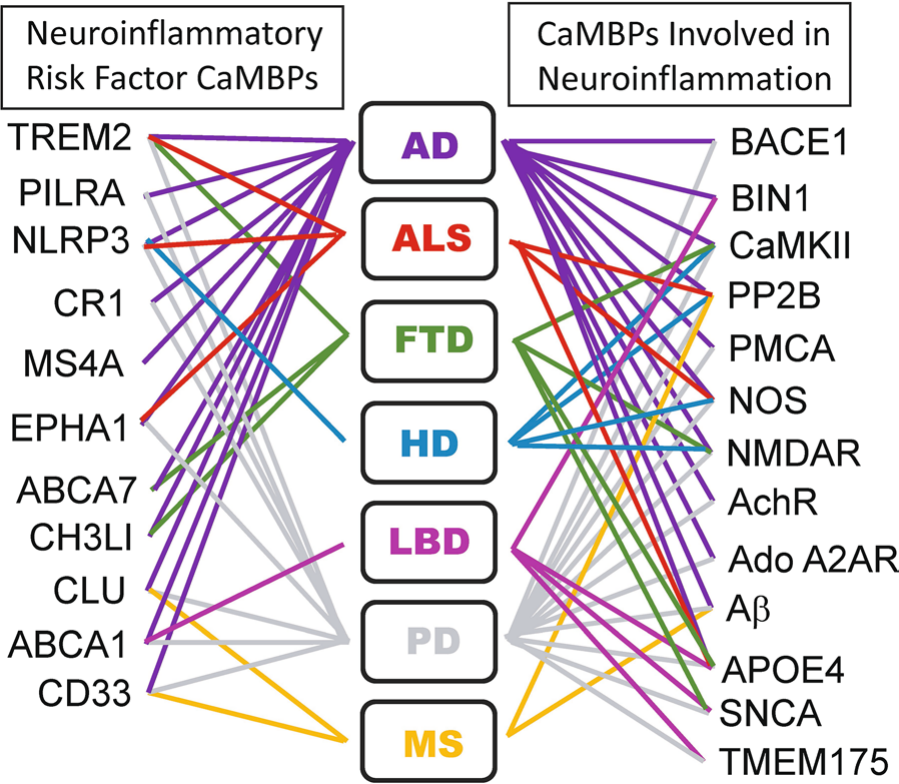
**Calmodulin binding proteins linked to neuroinflammation in specific neurodegenerative diseases**. See text for details

Previously, a central role for CaM and its CaMBPs has been shown for all phases of AD [42–44]. Here, we examined proteins identified by others that are critical to neuroinflammation and other neurodegenerative events involved not only in AD but also in HD, LBD, PD, FTD, MS and other neurodegenerative diseases [18, 19, 22, 50]. The presence of presumptive CaMBDs were identified in 11 proteins linked to neuroinflammation (ABCA7, CD33, CH3LI, CLU, CR1, EPHA1, MS4A4E, MS4A6A, NLRP3, PILRA, TREM2) (Fig. 3). Each of these proteins is linked to one or more neurodegenerative diseases (Fig. 4). For example. apolipoprotein E (APOE) is a neuroinflammatory risk factor CaMBP for AD, ALS, FTD, LBD and PD but not HD or MS. APOE has also been linked to BD, a family of neurodegenerative disorders clinically known as the neuronal ceroid lipofuscinoses (NCLs), in which CaMBPs have been predicted to play a significant role [25, 52]. In addition, there is strong genetic overlap of the NCLs with AD, PD, and FTD [2, 11, 14, 16, 49, 62]. In contrast, the CaMBP CLU is linked to AD, PD and MS. The role of TREM2, which is involved in AD, ALS, FTD and PD, has been recently reviewed [28]. Thirteen other previously identified CaMBPs, not classed as risk factors, have been associated with neuroinflammation in a diversity of neurodegenerative diseases: BACE1, BIN1, CaMKII, PP2B, PMCA, NOS, NMDAR, AchR, Ado A2AR, Aβ, APOE, SNCA, TMEM175 [5, 6, 8, 9, 12, 13, 21, 23, 29, 34, 36, 42, 44, 67] (Fig. 4). These CaMBPs also show a significant degree of overlap with different diseases. For example, while the CaMBP BACE1 functions in AD and PD, PP2B is associated with AD, ALS, HD, PD and MS. In total, two dozen proven or potential CaMBPs have a central involvement in neuroinflammatory events underlying multiple major neurodegenerative diseases. While many of these proteins are shown to be involved in neuroinflammation in multiple neurodegenerative diseases, this likely is underestimate that can only be clarified as more risk factor and other neuroinflammatory proteins are examined in a greater diversity of neurodegenerative events and diseases.

### The therapeutic potential of targeting calmodulin binding proteins in neurodegenerative disease

CaM and CaMBP based therapies have been used for decades and recently there has been an uptick not only in their use but in new approaches to using them. Numerous CaM targeting pharmaceuticals have been proven to be safe and effective for use. Both novel and traditional CaM antagonists (e.g., (trifluoperazine, tamoxifen) have been successfully used to treat a diversity of cancers including pancreatic cancer and cancer-dependent events such as angiogenesis [30, 66]. The immunosuppressant drug FK506 (Tacrolimus), which is an inhibitor of the classic CaMBP calcineurin, has long been used to prevent organ rejection after transplant surgery (e.g., [57]). In multiple studies, inhibition of calcineurin with FK506 have been proven to reduce plaque burden, restore memory deficits and reduce the incidence of dementia in humans and mouse models [24, 57]. The treatment of a Huntington’s mouse model (R6/2) with a peptide derived from the CaM sequence resulted in neuroprotection apparently through the inhibition of CaM binding to the huntingtin protein [15].

The importance of neuroinflammation in neurodegenerative disease is emphasized by the recent increased focus on anti-inflammatory treatments in dozens of ongoing clinical trials [10]. Many of these investigations focus on targets that are known CaMBPs including Aβ, tau, PP2B, NMDAR and AchR. Add to this, several targets are presumptive CaMBPs with identified binding motifs that are also in clinical trials: TREM2, APP and α-Syn. In addition, there are Phase 2 clinical trials focusing on the herbal remedy curcumin, which has been shown to bind to CaM. The flavonoid quercetin was identified in the 1980s as a CaMBP and a Phase 2 clinical study using it and a tyrosine kinase inhibitor is in the works. Hopefully if researchers find some success with any of these potential pharmaceuticals they will examine the specific role of CaM-binding in drug efficacy.

A review by Nassal et al. [40] points to ways a specific CaMBP, like CaMKII, can be targeted effectively to achieve a meaningful result. Since CaMKII is an important CaMBP in neuroinflammation and neurodegeneration, this example is appropriate with the essence of the information applicable to other CaM/CaMBP targets involved in neurodegeneration (e.g., [17, 43, 44]). Pharmaceuticals can be chosen that target a specific state of the CaMBP. KN-93 is an allosteric inhibitor of CaM binding CaMKII in its inactive state while AS105, GS-680, and RA306 are ATP-competitive inhibitors of activated CaMKII [40]. CaMKIItide and other peptide inhibitors (e.g., CN19o) are useful but issues of delivery and bioavailability exist. It is possible to overcome challenges in delivery via viral gene delivery and the use of nanoparticles [40]. Wang et al. [61] have used polysialic acid-based micelles to effectively cross the blood–brain barrier to deliver a CaM antagonist (DY-9836) for the treatment of vascular dementia. Their CaM inhibition research on the treatment of vascular dementia and bilateral carotid artery stenosis led to cognitive improvements possibly via the inhibition of nitric oxide overproduction (i.e., nitrosative stress) and inflammasome activation events involving the CaMBPs calcineurin and CaMKII. Finally, RNA interference, antisense oligonucleotides, small interfering RNA, and miRNAs offer alternative approaches to preventing CaMKII function [40]. These studies support the concept of targeting CaM and its binding proteins and provide multiple approaches for doing so. The goal now is to develop therapies that target specific CaMBPs linked to critical common events in neuroinflammation.

## Conclusions

Previous research, coupled with the analysis carried out here using the Calmodulin Target Database, has identified at least two dozen proven or putative CaMBPs that are central to neuroinflammation in multiple neurodegenerative diseases. Each CaMBP contains two or more binding domains with one or more classical binding motifs further supporting the binding of these proteins to CaM. Importantly, multiple pharmaceuticals targeting CaM and CaMBPs have been successfully used to treat several diseases indicating that clinical trials targeting neurodegenerative calmodulin binding proteins could be initiated immediately.

## Data Availability

All data generated or analyzed during this study are included in this published article.

## Abbreviations

ABCA1: ATP-binding cassette subfamily A member 1
ABCA7: ATP-binding cassette subfamily A member 7
AD: Alzheimer’s disease
ALS: Amyotrophic lateral sclerosis
BD: Batten disease
CaM: Calmodulin
CaMBD: Calmodulin binding domain
CaMBP: Calmodulin binding protein
CD33: Myeloid cell surface antigen CD33
CH3L1/YKL-40: Chitinase-3-like protein I
CLU: Clusterin
CR1: Complement receptor type 1
EPHA1: Ephrin type-A receptor 1
FTD: Frontotemporal dementia
HD: Huntington’s disease
LBD: Lewy body dementia
MS: Multiple sclerosis
MS4A: Membrane-spanning 4-domains subfamily A
NCL: Neuronal ceroid lipofuscinosis
NLRP3: NACHT, LRR and PYD domains-containing protein 3
PD: Parkinson’s disease
PILRA: Paired immunoglobin like type 2 receptor alpha
TREM2: Triggering receptor expressed on myeloid cells 2
